# Grand Challenges in Cancer Endocrinology: Endocrine Related Cancers, an Expanding Concept

**DOI:** 10.3389/fendo.2013.00141

**Published:** 2013-10-08

**Authors:** Antonino Belfiore, Claire M. Perks

**Affiliations:** ^1^Endocrinology, Department of Health Sciences, University Magna Graecia of Catanzaro, Catanzaro, Italy; ^2^IGFs and Metabolic Endocrinology Group, Faculty of Medicine, Southmead Hospital, University of Bristol, Bristol, UK

**Keywords:** endocrine related cancers, endocrine oncology, hormones and cancer, thyroid cancer, breast cancer, prostate cancer

The term Endocrine Related Cancers classically includes a group of sex steroid responsive cancers, such as cancers of the breast, endometrium, prostate, and testis, but also other cancers such as thyroid and ovary cancers that are responsive to pituitary hormones (1). Based on this widely accepted concept, at least 35–40% of newly diagnosed cancers fall into this definition (2). This concept has been useful for its clinical implications, and has lead to the use of anti-estrogenic/anti-androgenic treatments for sex hormone responsive cancers, as well as to thyroid-stimulating hormone (TSH) suppressive therapy with l-thyroxine (l-T4) for differentiated thyroid cancer. Moreover, several studies have tried to identify physiological conditions, such as early menarche, late menopause, age of first pregnancy, and number of pregnancies, prolonged lactation, and obesity, that may all affect sex steroids availability/exposure and modulate cancer risk. Another series of studies have identified germline polymorphisms, which are linked to the modulation of cancer risk through the genetic control of serum hormone levels or target tissue responses (3). Finally, other studies have addressed the possible effects of the exposure to exogenous sex hormones, such as estroprogestins (4) or postmenopausal estrogen replacing therapy (5) or endocrine disruptors (6). While all these studies have provided useful information for cancer prevention and treatment, our understanding of how genetic traits relevant to hormone action, control and interaction with physiological conditions, hormonal supplementations, and environmental factors is still limited. Another grand challenge is represented by the possibility to achieve effective and risk-free chemoprevention for subjects predisposed to individual endocrine related cancers, as exemplified by the use of tamoxifen and other agents in breast cancer predisposed subjects (6).

In recent years, several studies have challenged this classical view of endocrine related cancer. We now summarize recent data, which should be taken into account to reformulate the term “endocrine related cancer” in a more expanded way.

## Sex Steroid Hormones and Cancer: A Broad View

### Non-classical effects elicited by classical sex steroid receptors and non-classical estrogen receptors

#### Non-classical effects activated by androgen and estrogen receptors

It is well-established that a small proportion of canonical sex steroid receptors are located at the cell membrane. Upon cell stimulation by either estrogens or androgens, they form multiprotein complexes at the cell membrane that activate the Src/ERK/PI3K pathways (7). This pathway is named MISS (membrane-initiated steroid signaling) and may be upregulated in cancer cells for a number of reasons, among which increased location of sex steroid receptors at the cell membrane and increased expression of adaptor proteins, which favor the formation of multiprotein complexes containing Src (7). The activation of the Src/ERK/CREB pathway by MISS may enhance the activation of IGF system in prostate cancer (8). Intriguingly, MISS are scarcely inhibited by classical anti-hormone therapy, which preferentially blocks the genomic pathway. The occurrence of MISS in cancer should be more widely investigated as a potential new target to prevent resistance to anti-hormone therapy.

#### Non-classical receptors of steroid hormones involved in cancer

While classical estrogen receptors (ERα and ERβ) may elicit both canonical genomic effects and MISS, a member of the seven-transmembrane G protein-coupled receptor family, GPER, may also mediate both rapid and transcriptional events in response to estrogens, especially in cancer cells (9). GPER is expressed in both ER-positive and ER-negative breast carcinoma cells, and in a variety of other cancer cells including endometrial, choriocarcinoma, ovarian, and thyroid cancer cells (10). Accumulating evidence supports the notion that GPER may contribute to carcinogenesis and to resistance to anti-estrogen therapy. Noteworthy, the partial ER antagonist tamoxifen behaves as a GPER agonist (11) suggesting that conventional anti-estrogens may contribute to clonal selection of cancer cells. In ER-negative and tamoxifen-resistant breast cancer cells, significant cross-talk exists between both EGF and IGF pathways and GPER expression and signaling (10). GPER expression in cancer, ER/GPER cotargeting by novel inhibitors, and GPER cross-talk with IGF axis need to be further explored (10, 12). Interestingly, endocrine disruptors, such as Bisphenol A (13) or Cadmium, may induce estrogen like effects via GPER (14).

### Metabolic disorders and cancer: the role of “metabolic hormones” in cancer related tumors

#### Insulin, a new cancer related hormone

Overwhelming evidence now indicates that both obesity and type 2 diabetes mellitus (T2DM) are associated with up to two to threefold increased risk for various malignancies (15). Insulin resistance and compensatory hyperinsulinemia, present in these two disorders, appear to be the major determinants of this increased cancer risk (16) and are also associated to poor cancer prognosis. Although the underlying mechanisms are not fully clarified, two relevant factors are represented by the high level of circulating insulin and the biased insulin action in the presence of insulin resistance (17). A third factor is represented by insulin receptor (IR) overexpression by most cancers (17). Noteworthy, cancer cells overexpress the isoform A of IR (IR-A), which is predominantly expressed in embryo and fetal tissues, where it contributes to insulin and IGF-II mediated growth and development (18).

Although the degree of insulin resistance has a genetic basis, it is greatly modulated by diet and lifestyle. In fact, the rising occurrence of obesity and T2DM in western countries, and in the developing world, is causally associated with changing diet and lifestyle. Therefore, insulin resistance has become a major preventable cancer risk factor (19).

#### Chronic drug therapies and insulin resistance

In this context, it is increasingly recognized that a variety of chronic drug therapies, including oncologic treatments may cause or worsen insulin resistance. Whether they affect cancer risk or cause resistance to oncologic treatment should be addressed by future studies.

For instance, antipsychotic drugs can induce weight gain, insulin resistance, and other components of the metabolic syndrome (20). Some antidepressants, such as noradrenergic and tricyclic antidepressants, and antiepileptic drugs may also cause insulin resistance (21). Conventional chemotherapy, such as cisplatin-based chemotherapy, may also be associated with increased visceral adipose tissue, insulin resistance, and dyslipidemia (22). Conversely, IGF-I/Insulin signaling is associated with cisplatin resistance (23).

Moreover, anti-androgen and anti-estrogen therapies may be associated with worsening of insulin resistance. Anti-androgen therapy, besides being associated with well-established complications of male hypogonadism, is also associated with the development or worsening of insulin resistance and T2DM (24). A subtle consequence of chronic hyperinsulinemia in these patients could be cancer growth promotion and resistance to anti-androgen treatment. This possibility is presently underscored and deserves to be further explored. With regard to breast cancer patients receiving anti-estrogen therapies, contradictory data exist concerning patients using tamoxifen, although they usually present with liver steatosis (25), a well-known complication of insulin resistance. Moreover, insulin resistance is a common finding in patients treated with aromatase inhibitors (26).

Development of insulin resistance is a major side effect of cancer target therapy with IGF-IR blocking agents and may contribute to the dismal results of clinical trials employing these agents (19).

Development of insulin resistance in the course of cancer treatment should be now considered a factor that may significantly interfere with efficacy of treatment itself. We need, however, a new strategy to predict the individual risk of insulin resistant patients, including those receiving chronic therapies with drugs that worsen insulin resistance, not only in terms of metabolic complications, but also in terms of cancer risk or cancer resistance to therapy. The use of large-scale omics analyses (e.g., metabolomic, proteomic, transcriptomic, and lipidomic) may perhaps help to identify such new strategies.

#### Antidiabetic therapy and cancer

Antidiabetic drugs may modulate insulin sensitivity and/or circulating insulin levels. Insulin sensitizers, like metformin are associated with decreasing insulin levels, while administration of insulin secretion modulators or of exogenous insulin is often associated with non-physiological circulating insulin levels. Insulin analogs, which are now widely used in diabetes therapy, may elicit biased insulin effects at cellular levels (27). Epidemiological studies addressing the link between the use of specific insulin analogs and cancer risk are so far inconclusive and face various interfering factors, including the heterogeneity of the diabetic population (28). Also, preclinical studies with insulin analogs have not reached uniform conclusions. We also need more studies exploring the safety of specific antidiabetic drugs, such as PPARγ agonists and GPL-1 analogs and DPP4 inhibitors. Again, the use of large-scale omics analyses may hopefully be helpful in this context.

#### A cancer role for other metabolic hormones?

Accumulating evidence indicate that hormones secreted by non-classical endocrine organs may affect cancer risk through both direct and indirect mechanisms. Indirect mechanisms often act through the modulation of insulin secretion and sensitivity, as well as sex hormones secretion and metabolism. Leptin and adiponectin are two adipokines extensively studied in relationship to cancer (29).

Obesity is characterized by leptin resistance and increased leptin levels, and by reduced levels of circulating adiponectin. Receptors for both adipokines are present in cancer cells. Leptin may promote cancer both directly and by increasing aromatase expression and estrogen production (30). In contrast, circulating concentrations of adiponectin are inversely related to increased risk for a variety of malignancies. Adiponectin may also act directly on cancer cells but may also contribute to ameliorate insulin sensitivity and decrease circulating insulin levels (29).

Recently, a complex relationship between bone metabolism with glucose metabolism and sex steroid metabolism has been found. For instance, an osteoblast secreted protein, osteocalcin, affects insulin production and sensitivity and testosterone production (31). Moreover, low level of the steroid hormone vitamin D, is also implicated in worsening of insulin resistance and increased aromatase activity (31).

How this complex interplay between various metabolic hormones can be integrated in the concept of hormone sensitive cancer is still undefined. Moreover, how and to what extent this network may be modulated by diet and lifestyle or by pharmacological interventions in order to reduce cancer risk is still an open question.

## The Thyroid Model in Endocrine Oncogenesis

Thyroid gland function and trophism are mainly regulated by TSH, which is the key player of thyrocyte differentiation and proliferation. TSH has a well-known promoting role for differentiated thyroid cancer metastases, and TSH suppressive therapy with l-thyroxine, a well-established therapy in the postoperative management of differentiated thyroid cancer. However, to exert its maximal mitogenic and protumorigenic effects, TSH requires concomitant ligand-activated tyrosine kinase receptor signaling (32). Activation of the insulin/IGFs signaling pathway is especially important (32, 33). These observations open the way to possible clinical implications in the management of differentiated metastatic thyroid cancer. First, in order to avoid the possible side effects of subclinical hyperthyroidism induced by suppressive L-T4 doses, new modalities to block TSH can be envisaged. Second, TSH inhibition could be associated with inhibition of the insulin/IGF pathway.

While the promoting effect of TSH on metastatic cancer is well-established, only recently studies have observed an increased risk of thyroid cancer in subjects with serum TSH concentrations in the upper normal range (34). These observations are consistent with evidence that thyroid cancer risk is increased in endemic goiter iodine deficient areas (35). Iodine deficiency, which makes the thyroid gland more responsive to the mitogenic effect of TSH, has long been suspected to be associated with follicular thyroid cancer. However, the relationship between iodine intake and the risk of thyroid cancer is probably non-linear and further studies are needed to clarify it. Intriguingly, increased iodine intake may be associated with an increase of autoimmune thyroiditis, which in turn, may cause subclinical hypothyroidism and raised serum TSH (36). Moreover, Graves’ disease, an autoimmune disorder characterized by the presence of TSH receptor stimulating antibodies (TSHR-Abs) has been found to be associated with increased thyroid cancer risk and thyroid cancer aggressiveness (37). TSHR-Abs, however, are heterogeneous with regard to serum levels, binding characteristics to the TSHR, and biological effects in different thyroid cancer patients.

Thyroid follicular cancer cells express both classical (ERα and ERβ) and non-classical (GPER) estrogen receptors, which may help explain why thyroid cancer is more frequent in females. Moreover, thyroid cancer cells and stem-like thyroid cells also overexpress the IR and IGF-IR (38), which may be involved with the increased thyroid cancer risk with obesity and insulin resistance. Preliminary epidemiological data suggest that obesity may also be associated with a less favorable clinicopathologic profile of thyroid cancer. Large multi-center studies are still needed, and the possible involvement of adipokines, like leptin and adiponectin, should be addressed by future studies.

Intriguingly, the incidence of differentiated thyroid cancer is increasing more rapidly than that of other cancers in the United States (39) and in many countries worldwide, with no obvious explanation. It is likely that thyroid cancer will continue to offer a fascinating model of endocrine related cancer to study the complex relationship between cancer risk and TSH, insulin/IGFs and estrogens, but also the interplay of these hormonal factors with iodine intake, thyroid autoimmunity, and other environmental factors like endocrine disruptors.

### Models of carcinogenesis: What place for hormones?

The increased awareness that hormones may modulate the risk and progression of most cancers poses the question of how hormone action fits into a general model of carcinogenesis. It is generally accepted that hormones may enhance the effects of mutagens or of spontaneous mutations by accelerating proliferation and inhibiting apoptosis of mutated cells (3). However, there is increasing evidence that epigenetic changes have an important role in the carcinogenetic process. Indeed, hormones do cause epigenetic changes, especially at critical ages, such as pre- and perinatal life (40). Actually, epigenetic changes may explain cancer resistance to endocrine therapy (41). Recently, the stem cell model of carcinogenesis has been extended to solid tumors (42). Hormones, including insulin/IGFs, do affect stem cell biology and stem cell carcinogenesis (43). Unbalanced hormone production, favored by nutritional and/or various environmental factors, may expand the stem cell pool at perinatal age, thus affecting cancer risk at later ages (44). A better understanding of these mechanisms may open the way to effective cancer prevention. Hopefully, advances in all these areas will contribute to develop an improved general model of carcinogenesis.

## Conclusion and Perspectives

Most cancers appear “hormone sensitive” at least at some stages of their development and/or progression. It is clear now that the relevant hormones include also a typical metabolic hormone such as insulin, and possibly other metabolic hormones. We need better definitions, and better assessment of what hormone sensitivity means for individual cancer histotypes. This should bring about a consistent shift in our concept of “hormone sensitive” cancer, and has several relevant implications. New models of carcinogenesis should then incorporate mechanisms of hormonal action in cancer initiation and progression and should identify critical ages for hormonal carcinogenesis. As far as cancer treatment is concerned, the possibility exists that most cancer histotypes will be considered amenable to some form of hormone treatment in the near future.
